# Human Papillomavirus-16 DNA Quantitation Differentiates High-Grade Anal Neoplasia

**DOI:** 10.3390/ijerph15081690

**Published:** 2018-08-08

**Authors:** Melissa Agsalda-Garcia, Tiffany Shieh, Eleanore Chuang, Nicholas Loi, Cris Milne, Rui Fang, Eunjung Lim, Jeffrey Killeen, Bruce Shiramizu

**Affiliations:** 1 Hawaii Center for AIDS, Honolulu, HI 96813, USA; magsalda@hawaii.edu (M.A.-G.); tshieh@hawaii.edu (T.S.); eleanore@hawaii.edu (E.C.); NLoi@hawaii.edu (N.L.); cmilne@hawaii.edu (C.M.); 2 Department of Tropical Medicine, Medical Microbiology, and Pharmacology, John A. Burns School of Medicine, University of Hawaii, Honolulu, HI 96813, USA; 3 Complementary and Integrative Medicine, John A. Burns School of Medicine, University of Hawaii, Honolulu, HI 96813, USA; fangr@hawaii.edu (R.F.); lime@hawaii.edu (E.L.); 4 University of Hawaii Cancer Center, Honolulu, HI 96813, USA; Jeffrey.Killeen@hawaiilabs.com; 5 Pathology Department, John A. Burns School of Medicine, University of Hawaii, Honolulu, HI 96813, USA

**Keywords:** human papillomavirus, HPV, HIV/AIDS, men who have sex with men, MSM, anal cancer, cytology, dysplasia

## Abstract

*Background:* Due to their higher rates of anal dysplasia/cancer, human immunodeficiency virus (HIV)-positive individuals are recommended to undergo anal dysplasia screening, which consists of anal cytology (AC) and high resolution anoscopy (HRA) with anal biopsy (AB) after abnormal AC result. However, AC variability limits its usefulness. Our objective was to evaluate human papillomavirus (HPV)-16 DNA quantitation as part of the screening algorithm. *Methods:* HPV-16 was detected in AC specimens from 75 HIV-positive participants using quantitative real-time polymerase chain reaction. AB results were available from 18/44 patients who had abnormal AC. Statistical tests included Mann-Whitney U, Kruskal-Wallis, receiver operating characteristic (ROC) analysis and Kappa coefficient tests. *Results:* HPV-16 copy numbers differed significantly across AC (*p =* 0.001) and AB grades (*p* = 0.009). HPV-16 ≥ 65 copies/cell predicted high-grade AB (*p =* 0.04). Using this cut-off in comparison to AB, it had better specificity (1.00) than AC (0.75) and specificity (0.77) than qualitative HPV-16 detection (0.38). Also, the Kappa coefficient of the cut-off (κ = 0.649) was higher than AC (κ = 0.557) and qualitative HPV-16 detection (κ = 0.258) to AB. *Conclusion:* Higher HPV-16 copy numbers corresponded to higher AC and AB grades, suggesting the importance of HPV burden on disease stage. Furthermore, HPV-16 ≥ 65 copies/cell distinguished high-grade disease and demonstrated better sensitivity, specificity, and agreement with AB than AC or qualitative HPV-16 detection. These results support the potential use of HPV quantitation in conjunction with AC in anal dysplasia screening.

## 1. Introduction

Human immunodeficiency virus (HIV)-infected individuals are at a higher risk for developing anal dysplasia and cancer, particularly men who have sex with men (MSM) [1,2,3,4]. Histopathological changes in the squamous epithelium of the transitional zone manifest from human papillomavirus (HPV) infection and lead to anal squamous intraepithelial lesions (ASIL) ranging from low-grade to high-grade SIL (LSIL, HSIL) which represents the spectrum of anal dysplasia pathologies including those associated with high-risk HPV types, with HPV-16 accounting for the majority of anal cancer cases [2]. Due to their increased risk for anal dysplasia/cancer, HIV-infected individuals are recommended to undergo regular anal cytology (AC) screening [5]. AC grades are classified as atypical squamous cells of undetermined significance (ASCUS), atypical squamous cells but cannot exclude high-grade (ASC-H), low-grade squamous intraepithelial lesions (LSIL), or high-grade squamous intraepithelial lesions (HSIL). If an individual is found to have an abnormal AC, he/she is recommended to undergo more invasive high resolution anoscopy (HRA), the current gold standard in ASIL and anal cancer diagnosis. During HRA, biopsies are taken from areas with apparent morphological changes in tissue. Abnormal anal biopsy (AB) are classified using the lower anogenital squamous terminology (LAST) [6] as either low-grade squamous intraepithelial lesions (LSIL) or high-grade squamous intraepithelial lesions (HSIL).

Currently, treatment is recommended only for those with high-grade disease. Because low-grade disease is not typically treated, screening that identifies LSIL prior to HRA and AB could reduce the number of invasive procedures that appear unnecessary in retrospect. Another factor that influences AC results is its inherent sensitivity and specificity as a screening tool [7]. Although AB during HRA is considered the current gold standard, it is also subject to variability in sensitivity and specificity, which highlights the need for improved screening with consistent sensitivity and specificity for high-grade lesions to prevent unnecessary HRAs, ABs, and other interventions [7,8,9,10].

Since HPV-16 is the most prevalent high-risk HPV genotype in anal dysplasia/cancer and is associated with high-grade lesions [11,12], the objective of the study was to determine if HPV-16 DNA quantitation could improve the screening algorithm in distinguishing low-grade disease from high-grade disease. Ultimately, results of the study could decrease the number of HRAs and ABs performed for low-grade disease. We hypothesized that higher HPV-16 DNA copy numbers would correspond to high-grade AC and AB. In this study, we found that HPV-16 DNA copy numbers differed according to AC and AB grades and that a cut-off value of 65 HPV-16 copies per cell, as determined by receiver operating characteristics (ROC) analysis, differentiated low-grade from high-grade disease and provided high sensitivity and specificity. These findings support the utility of adding HPV-16 quantitation to the anal dysplasia screening algorithm.

## 2. Materials and Methods

### 2.1. Patient Enrollment and Specimen Collection

For the Hawaii Center for AIDS RMATRIX Pilot Project RM004, 75 HIV-positive patients enrolled and provided consent under a protocol approved by the University of Hawaii Institutional Review Board (CHS #21953). AC specimens were collected in ThinPrep Collection vials during one study visit for routine healthcare maintenance and high-risk HPV (hr-HPV) assessment. Abnormal AC results were reported as ASCUS, ASC-H, LSIL, or HSIL. Participants were followed up after the AC results were available as part of routine medical care, and HRA was recommended to those with abnormal AC. If HRA was performed, and biopsies were obtained, biopsy results from participants who gave consent were available for the study. AB pathologies were reported as normal, LSIL, or HSIL.

### 2.2. Anal hr-HPV

DNA was extracted from AC specimens, using the Machery-Nagel NucleoSpin Tissue XS Kit, and quantitated on a NanoDrop 2000 instrument. Specimens were analyzed for HPV-16 genotype by quantitative real-time PCR (qRT-PCR), using primers and probes targeting the E6/E7 oncogenic region [13] and β-globin for the housekeeping gene. Analyses were completed using SDS 2.3 software (Applied Biosystems, Foster City, CA, USA). Standard curves were derived from ten-fold serial dilutions of β-globin plasmid and HPV-16 plasmid—p1203 PML2d HPV-16 was a gift from Peter Howley (AddGene #10869)—at calculated quantities from 10 copies to 1 million copies. Controls included DNA from SiHa cells (American Type Culture Collection ATCC^®^ HTB-35™), positive for HPV-16; and water. All qRT-PCR assays were performed in triplicate, using TaqMan™ Gene Expression Master Mix (Thermo Fisher Scientific, Waltham, MA, USA). Copy numbers of each target gene were calculated based on the standard curve, and HPV-16 copy numbers per cell were determined.

### 2.3. Statistical Analysis

The data were summarized by mean and standard deviation (SD) for continuous variables such as age and HPV-16 copy numbers; number of participants and percentage for the categorical variables such as gender, smoking, and race. Two-sample *t*-test or Mann-Whitney U test was used for continuous variables, such as age and CD4 count.

Chi-square or Fisher’s exact test was used for the categorical variables, such as gender, smoking, and race. Mann-Whitney U test was used to determine whether qualitative HPV-16 detection differed between positive and negative AC or AB results. Kruskal-Wallis test was performed to assess statistical significance of HPV-16 copy numbers relative to AC or AB grade. ROC analysis was used to select the choice of a cut-off point for HPV-16 copy number to differentiate low-grade and high-grade AB. Sensitivity, specificity and Cohen’s Kappa coefficient were calculated to compare the following screening assays to the gold standard of HRA with AB: AC, qualitative HPV-16 detection, and HPV-16 copy number at a cut-off of 65 copies per cell. Negative and low-grade disease grades were combined since clinical recommendations for both would typically be medical follow-up without treatment. All analyses were performed using SAS 9.4 and GraphPad Prism, and a *p*-value < 0.05 was considered to be statistically significant.

## 3. Results

**Figure 1:**
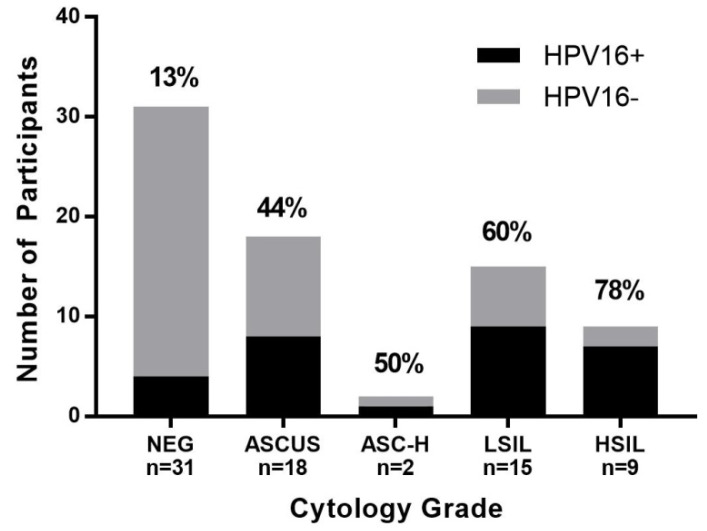
Detectable HPV-16 across anal cytology (AC) grades. Number of participants with positive or negative HPV-16 detection by cytology grade. Percentages depict HPV-16+.

**Figure 2:**
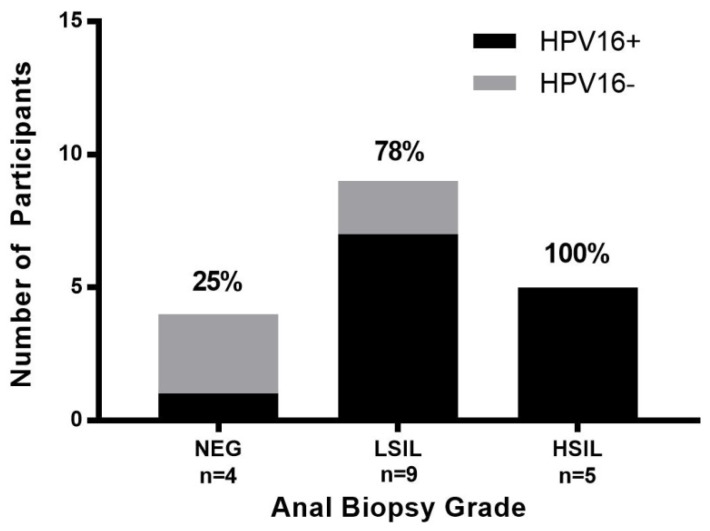
Detectable HPV-16 across anal biopsy (AB) grades. Number of participants with positive or negative HPV-16 detection by AB grade. Percentages depict HPV-16+.

**Figure 3:**
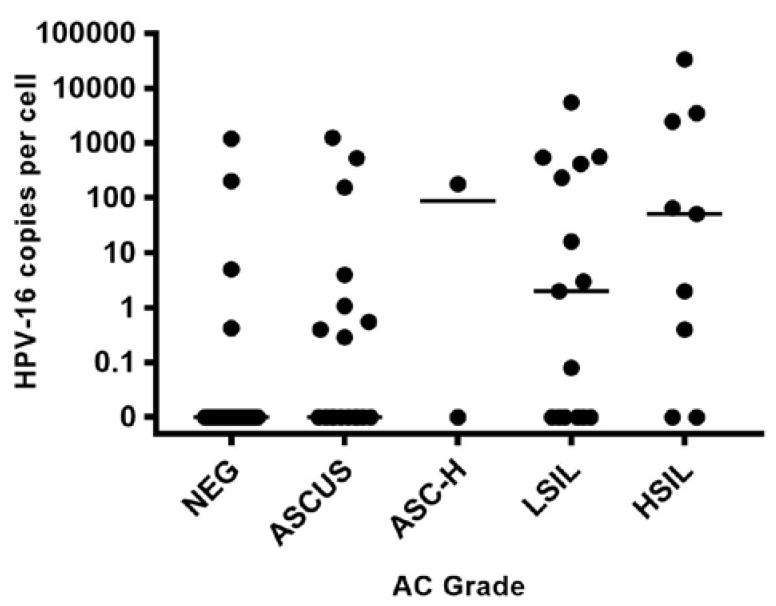
HPV-16 copy number by AC grade. HPV-16 copy numbers per cell of participants separated by AC grade.

**Figure 4:**
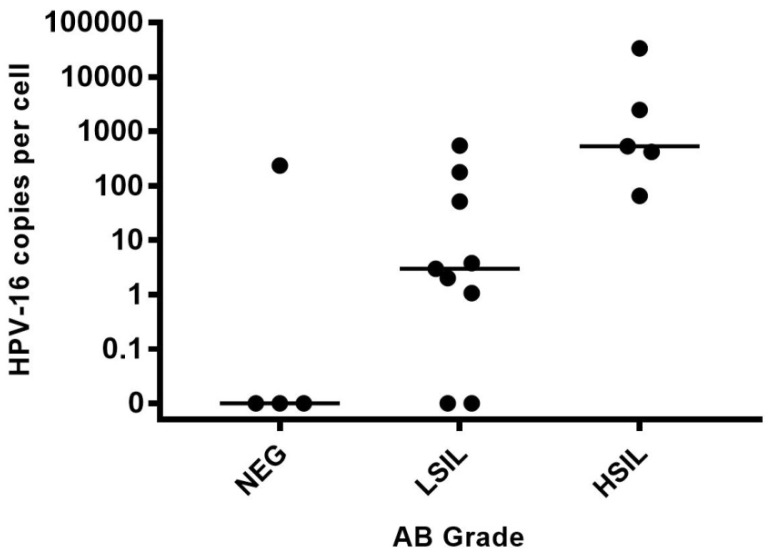
HPV-16 copy number by AB grade. HPV-16 copy numbers per cell of participants separated by AB grade.

A total of 75 HIV-positive participants were enrolled in the study. Overall, 67 (89%) were male with a mean age of 51 years. Forty-four (59%) had an abnormal AC, of which 25 (57%) had detectable HPV-16 DNA, a significantly higher rate than those with negative AC (19%), *p =* 0.005, Table 1. The percentage of participants with detectable HPV-16 DNA did not significantly increase with disease grade severity across AC and AB grades, Figure 1 and Figure 2. However, HPV-16 copy numbers were statistically different according to AC grade (*p =* 0.001) with the following medians (IQR): Negative 0 (0–0), ASCUS 0 (0–1.8), ASC-H 89 (0–178), LSIL 2 (0–417), and HSIL 51 (0.2–2991), Figure 3. A total of 18 participants completed an HRA with AB and HPV-16 copy numbers were also statistically different according to AB grade (*p =* 0.009) with the following medians (IQR): Negative 0 (0–117.5), LSIL 3 (0.54–114.5) and HSIL 530 (241–18018), Figure 4.

**Figure 5:**
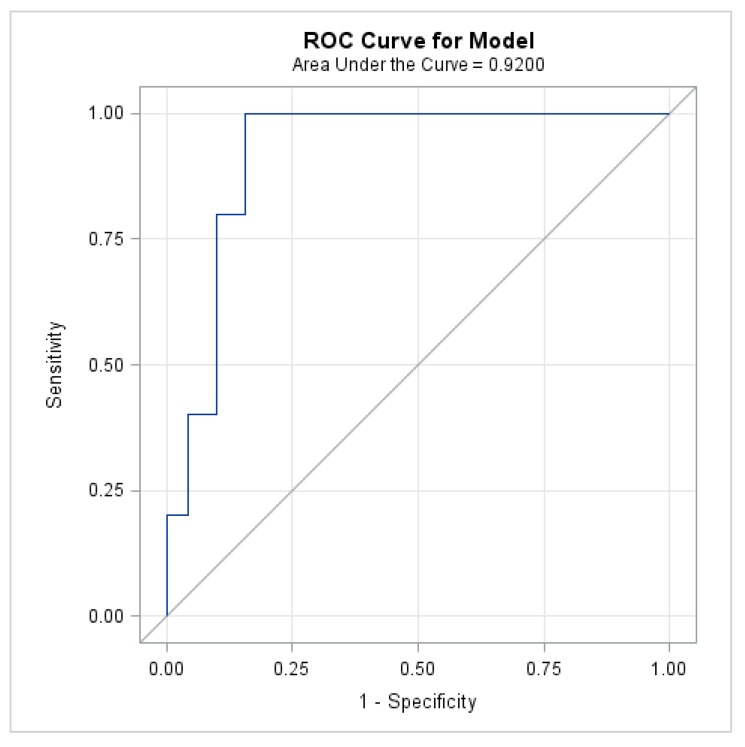
Receiver operating characteristic (ROC) curve used to determine high-grade squamous intraepithelial lesions (HSIL) prediction using HPV-16 copy number.

Since higher HPV-16 copy numbers exhibited an association with more advanced disease, ROC analysis was used to determine that HPV-16 copy number ≥65 copies per cell predicted HSIL with the area under the ROC curve (AUC) = 0.920, Figure 5.

When comparing the screening assays against the current gold standard of HRA with AB, we found that AC had lower sensitivity (0.75) with a reasonable specificity (0.86), qualitative HPV-16 had a better sensitivity (1.00) with a low specificity (0.38), and HPV-16 copy number cut-off at 65 copies per cell also had the same sensitivity as qualitative HPV-16 (1.00) but with increased specificity (0.77), Table 2. Cohen’s Kappa coefficient comparing AC and AB results was 0.557 (95% CI: 0.117, 0.998); however, there was higher agreement between AB results and HPV-16 copy number when the cut-off at 65 copies per cell was applied: 0.649 (95% CI: 0.31, 0.989), Table 2. The Kappa coefficient comparing qualitative HPV-16 detection and AB results was 0.258 (95% CI: 0.001, 0.515) which was lower agreement and specificity than for HPV-16 quantitation using the cut-off value of 65 copies per cell versus AB results, Table 2.

## 4. Discussion

In our study cohort, more than half (59%) of the participants had abnormal AC, which is consistent with other studies [14] that suggest HIV-positive individuals are at high risk for developing anal dysplasia. We also detected HPV-16 DNA in AC specimens from more than half (57%) of the participants with abnormal AC results, a significantly higher rate (*p =* 0.005) than for those with negative AC results (19%). Among all participants, HPV-16 was detected at a rate (41%) consistent with other studies suggesting that HIV-infected individuals are more prone to HPV infections, which contribute to their risk for anal dysplasia/cancer [15,16,17,18,19]. While there was no statistical significance in qualitative HPV-16 detection across both AC and AB grades, average HPV-16 copy numbers increased at higher AC and AB grades (*p =* 0.001 and *p =* 0.009 respectively). The failure to reach statistical significance for qualitative HPV-16 detection may be due to the small sample sizes that resulted after stratification into the various grades. Although AC had reasonable specificity (0.86) when compared to the current gold standard AB, it had a decreased sensitivity (0.75) which would miss the detection of some high-grade lesions. Anal dysplasia/cancer screening guidelines have generally been adapted from cervical cancer screening because of many similarities, such as association with HPV infection. Current cervical cancer screening includes qualitative HPV detection of oncogenic types in conjunction with cytology. We found that when compared to AB, the use of qualitative HPV-16 detection had high sensitivity (1.00) but a low specificity (0.38). The use of a HPV-16 cut-off value of 65 increased the specificity (0.77) to better differentiate grade of lesions, demonstrating that quantitative HPV-16 detection using the cut-off value of 65 copies per cell may predict high-grade disease and is in better agreement with AB (0.649) than AC (0.557) or qualitative HPV-16 HPV detection (0.258). Genotyping studies have shown that HPV-16 DNA is frequently detected in anal specimens and is associated with anal dysplasia/cancer; however, there is limited data about variation in HPV copy numbers across AC and AB grades [20,21]. Our findings suggest that HPV-16 quantitation may be useful in disease classification during screening for anal dysplasia/cancer.

Limitations of this study include the small number of patients who followed up with recommended HRA and AB after abnormal AC results. The small sample size may influence the HPV-16 cut-off value. In a planned follow-up study, patients will be encouraged to return for HRA with AB after abnormal AC results. The study also focused on only HPV-16 even though other high-risk HPV genotypes are associated with anal dysplasia/cancer. In addition, HRA was only performed on participants who had abnormal anal cytologies. Future research could include quantitation of other HPV genotypes such as HPV-18 as well as performing HRA on all participants regardless of anal cytology results.

## 5. Conclusions

Since HIV-positive individuals have a high risk of developing anal dysplasia/cancer they are recommended to undergo regular screening that consists of AC followed by an HRA with AB for abnormal AC. However, consistency of anal dysplasia/cancer AC screening results depends upon several factors such as sampling, operator error, and interpretation error [7]. The consequences of inconsistent AC screening include cases when high-grade lesions are not found during follow-up HRA and AB, the current gold standard. In the current study, higher HPV-16 DNA copy numbers corresponded to higher AC and AB grades. We also demonstrated that a cut-off value of 65 copies per cell distinguished low-grade from high-grade disease. Using this cut-off, we found better sensitivity and agreement between HPV-16 quantitation and AB than between AC and AB, and better specificity and agreement than between qualitative HPV detection and AB. These results emphasize the impact of HPV burden on anal dysplasia disease stage and suggest that HPV-16 quantitation may improve the current screening paradigm, which may help to decrease the number of unnecessary invasive HRAs with AB as well as missed detection of high-grade lesions.

## Figures and Tables

**Table 1:** Demographics. Characteristics of study participants.

	All Participants (*n* = 75)	Negative AC (*n* = 31)	Positive AC (*n* = 44)	*p*-Value
Age, mean (SD)	51 (10.6)	51 (11.9)	50 (9.7)	0.73
Gender, *n* (%) Male	67 (89)	28 (90)	39 (89)	0.99
Detectable HPV-16	31 (41)	6 (19)	25 (57)	0.005
CD4 nadir count, median	208	231	191	0.34
Smoking, *n* (%)				0.53
Current Smoker	19 (25)	6 (19)	13 (30)	
Past Smoker	36 (48)	17 (55)	19 (43)	
Never Smoked	20 (27)	8 (26)	12 (27)	
Race, *n* (%)				0.79
Native/Alaskan American	2 (3)	1 (3)	1 (2)	
African American	4 (5)	1 (3)	3 (7)	
Asian	11 (15)	4 (13)	7 (16)	
White	38 (51)	16 (52)	22 (50)	
Hawaiian/Pacific Islander	10 (13)	6 (19)	4 (9)	
More than One	10 (13)	3 (10)	7 (16)	

SD: Standard Deviation; AC: Anal Cytology.

**Table 2:** Sensitivities, specificities and Kappa coefficients of AC, qualitative HPV-16, and HPV-16 copy ≥65 vs. AB.

	Sensitivity	Specificity	Kappa (κ) (95% CI)
AC vs. AB	0.75	0.86	0.557 (0.117, 0.998)
Qualitative HPV-16 vs. AB	1.00	0.38	0.258 (0.001, 0.515)
HPV-16 copy ≥ 65 vs. AB	1.00	0.77	0.649 (0.031, 0.989)

CI. Confidence Interval.
